# Spatio-temporal distribution and environmental determinants of dengue vectors in Phnom Penh, Cambodia

**DOI:** 10.1371/journal.pntd.0013667

**Published:** 2025-10-29

**Authors:** Vincent Herbreteau, Pierre-Olivier Maquart, Sokeang Hoeun, Bros Doeurk, Florian Girond, Sébastien Boyer

**Affiliations:** 1 ESPACE-DEV, IRD, Univ Montpellier, Univ Antilles, Univ Guyane, Univ Réunion, Univ Perpignan et Univ Nouvelle-Calédonie, Phnom Penh, Cambodia; 2 Epidemiology and Public Health Unit, Institut Pasteur du Cambodge, Phnom Penh, Cambodia; 3 Institut de Recherche pour le Développement, UMR-247 EGCE IRD, CNRS, Université Paris SaclayGif sur Yvette Cedex, Gif-sur-Yvette, France; 4 Medical and Veterinary Entomology Unit, Institut Pasteur du Cambodge, Phnom Penh, Cambodia; 5 Communicable Disease Control Department, Ministry of Health, Phnom Penh, Cambodia; 6 Ecology and emergence of arthropod-borne Pathogens Unit, Department of Global Health, Institut Pasteur, CNRS, UMR2000, Paris, France; Instituto Oswaldo Cruz, BRAZIL

## Abstract

Dengue fever, one of the most widespread vector-borne diseases globally, is mainly transmitted by *Aedes aegypti* and *Ae. albopictus* mosquitoes. In Cambodia, dengue has been a recurrent public health challenge, with major outbreaks occurring in 1995, 2007, 2012, and 2019. The latter epidemic severely impacted the capital, Phnom Penh, yet the spatial and temporal dynamics of the two key vector species had not been studied in this urban context. This study aimed to investigate how the distribution of *Ae. aegypti* and *Ae. albopictus* is organized in the urban and peri-urban landscapes of Phnom Penh. Ovitraps were deployed every two months over a year in 40 pagodas randomly selected across Phnom Penh, chosen to ensure future replicability of the study. The larvae collected were reared to adulthood for accurate species identification. High-resolution satellite imagery (SPOT7) and daily rainfall data were used to analyze the surrounding environments through remote sensing techniques. The results revealed distinct spatio-temporal patterns for each species: *Ae. albopictus* was associated with peri-urban areas rich in vegetation and water, while *Ae. aegypti* predominated in highly urbanized and construction-dense environments. Spatial analysis using buffer zones (250 m, 500 m, 1000 m) around trapping sites confirmed that the use of pagodas as proxies for urban sampling is effective. These findings highlight the importance of monitoring these vector species, particularly as Phnom Penh continues to undergo rapid environmental transformation. The identification of simple, remotely sensed environmental indicators offer a valuable tool for predicting future outbreaks and guiding targeted vector control strategies. This study also provides a replicable methodological framework to assess the impact of urbanization and climate change on dengue vector distribution in Phnom Penh and similar urban settings.

## Author summary

Dengue fever is a major health concern in Southeast Asia, especially in Cambodia, where outbreaks repeatedly affect the population. The pathogen, the dengue virus, is transmitted by two main mosquito vector species, *Aedes aegypti* and *Aedes albopictus*. In this study, we explored where and when these mosquito species are most active in Phnom Penh, the rapidly growing capital city of Cambodia. By placing traps to collect mosquito larvae in 40 pagodas over a year and combining these data with satellite images and rainfall records, we identified differences about preferences for the two species distribution. *Ae. aegypti* was preferentially found in dense, urban areas, while *Ae. albopictus* preferred greener, peri-urban zones. Spatial analysis using multiple buffer zones (250 m, 500 m, 1000 m) confirmed these associations and demonstrated that pagodas serve as effective and replicable sampling proxies in urban settings. Also, this research provides a replicable framework for assessing mosquito vector dynamics in rapidly urbanizing environments. By linking remotely-sensed environmental indicators to vector presence, the study offers a scalable approach for predicting dengue risk and guiding targeted vector control strategies. These insights are particularly relevant for cities like Phnom Penh, where urban expansion and climate variability may significantly influence vector ecology and the epidemiology of dengue fever.

## Background

Dengue fever and dengue haemorrhagic fever are two of the most important mosquito-borne viral diseases of public health significance [1,2]. Dengue is affecting a growing number of countries: while only five countries reported dengue cases in the 1950s, more than 130 countries now report cases of dengue fever or dengue haemorrhagic fever [3,4]. This growth has been particularly worrying in recent years, when the highest numbers of cases have been recorded: 5.2 million in 2019 and 6.5 million in 2023, according to the World Health Organization [5]. Beyond the number of cases reported by healthcare systems, the impact is much greater, and modelling work carried out in 2013 estimated that more than 390 million people are infected yearly with dengue worldwide, of which approximatively 96 million manifest clinically [6,7]. More than half of the world population currently lives in regions where dengue transmission occurs, making this arbovirus one of the world’s most widespread [7]. The majority of the at-risk population currently lives in the Asia-Pacific Region (W.H.O., 2020).

In Cambodia, since the massive epidemic in 1995, accounting for more than 400 deaths, the number of cases has been monitored yearly and has continued to increase [8,9]. Major dengue epidemic outbreaks occurred in 2007 (39,618 cases with 396 deaths), 2012 (42,362 cases with 189 deaths) and 2019 (68,597 cases with 48 deaths) [10]. In 2018 and 2019, the capital Phnom Penh city was affected as never before, with respectively 9,445 cases in 2018 and 9,298 cases in 2019 [10]. The capital is a fast-developing city with a demographic growth rate of 3.2% per year between 2008 and 2019 (2.282 million inhabitants in 2019, including 627,646 new inhabitants in 11 years), associated with significant and rapid urban sprawl and infrastructure development. Indeed, the urban area has increased eightfold between 1973 and 2015 [11]. While the population of Phnom Penh initially expanded into agricultural lands, since 2016, urban sprawl has predominantly occurred through the filling of lakes and wetlands within the metropolitan area [11]. The population density is quite high (3,136 inhabitants per square kilometre) partially explaining the last outbreak in Phnom Penh in 2019: One hypothesis is that the rapid urbanisation of the city is conducive to the creation of multiple breeding sites in the urban centre. Urbanisation could indeed favour vector proliferation as it increases the number of potential breeding sites for mosquitoes, particularly dengue virus vectors [12].

The two main mosquito species responsible of the transmission of dengue viruses are *Aedes aegypti* (Linnaeus, 1762) and *Ae. albopictus* (Skuse, 1894) [13–15]. In Phnom Penh, despite surveys of dengue vectors undertaken in the 2000s [12,16–18] and although *Ae. aegypti* distribution, occurrence and genetics have been described [18], the presence of *Ae. albopictus* in the capital city was attested only recently [10]. Consequently, it is important to better describe the ecology and seasonality of both species to develop effective and cost-effective vector/disease control programs in the Cambodian capital. Based on this observation, this study aims to understand the geographical and temporal distribution of the two *Aedes* mosquito species, in order to highlight possible environmental factors that could determine their presence, while proposing a simple method based on remote sensing techniques that can be replicated in the future or in other cities to understand the impact of urbanization and environmental changes.

## Methods

### Ethic statement

This study was approved by the Cambodian authorities, specifically by the Ministry of Cults and Religions, under permit n°321/19, signed on 03 April 2019.

### Study area and design

Phnom Penh, the capital city of Cambodia, is located in the south-central part of the country, at the confluence of the Mekong, Tonle Sap and Tonle Bassac rivers. Phnom Penh municipality covers 290 square kilometres and is divided into 14 administrative divisions or “Khans” (districts). These sections are subdivided into 105 Sangkats (communes), further subdivided into 953 villages. Phnom Penh has a tropical wet and dry climate according to the Köppen climate classification [19]. The average temperature is 28.3°C, ranging from 22 to 35°C, with the lowest temperatures during the dry season between December and February and highest temperatures between March and May. The average annual total rainfall is 1,412mm with the highest precipitation in September and October. The driest months are from December to March.

This study was designed to be replicated in a few years to explore the dynamics and distribution of dengue vectors in Phnom Penh. Considering the rapid urbanization, we chose to trap mosquitoes in pagodas (the name given to Buddhist places of worship in Cambodia), which should be preserved in the face of the galloping urbanization facing the city in the coming years. They are distributed in the heart of the capital and in the surrounding agglomeration. Indeed, these religious buildings not only serve a cultural role (*i.e.,* religion) but also have a social purpose as educational facilities. Generally, within the pagoda compound, there is vegetation (trees, potted plants, etc.), small ponds (some natural), water containers (artificial ponds, concrete jars etc.), buildings, and animals.

The study was planned to last one year, so a compromise had to be made between the number of sites to be monitored to allow spatial analyses, the number of visits during the dry and rainy seasons, and the number of sites that can be investigated in one day by the entomological team. It was therefore agreed that 40 pagodas could be studied, with a visit every 8 weeks (*i.e.,* 6 times over one year for each pagoda). This periodicity allowed us to sample five pagodas a week, so that all 40 pagodas were sampled after 8 weeks. In each pagoda, mosquito larvae were collected in 5 different locations by leaving the traps for one week.

### Identification, mapping and selection of pagodas

**Fig 1:**
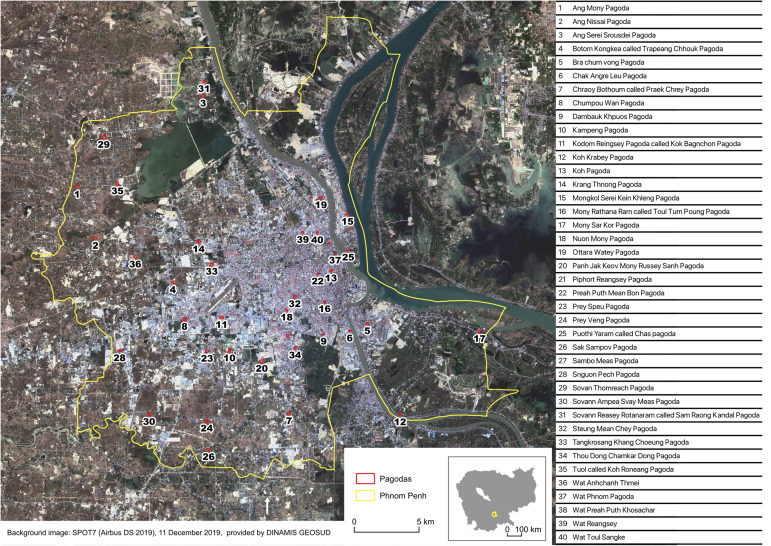
Location of the 40 selected pagodas in Phnom Penh, Cambodia.

In order to make a random selection of 40 pagodas from among all the existing pagodas in Phnom Penh, we first had to compile an exhaustive inventory and check their location. This identification and localization of the pagodas were done by querying available geographic databases, *i.e.,* Google Maps (https://www.google.com/maps) and OpenStreetMap (OSM: https://www.openstreetmap.org/). In Google Maps, 122 pagodas were identified in Phnom Penh, and 154 in OSM, with duplicates. Since OSM is an open-source database, we chose to check all the pagodas described in OSM, to remove duplicates, add missing pagodas, and complete their names in Khmer. We finally obtained the location of 141 pagodas. We also used OSM to precisely map, by photointerpretation, the compound of each pagoda (the compound outline and every building, tree and pond). We then used QGIS software [20] to import the cartographic elements we have created in OSM, with the QuickOSM plugin. We randomly selected 40 pagodas from the 141 identified in Phnom Penh, using QGIS, and these pagodas were the ones included in the study (Fig 1).

### Mosquito collection and identification

In each pagoda, five ovitraps were placed in total (four at each of their corners and one at its center) to collect mosquito larvae without filter paper inside in order to collect larvae instead of eggs. The ovitrap consists of a black plastic bucket filled with water. This type of trap is specifically designed to attract gravid *Aedes* females that will lay their eggs. After a week, the contents of the traps (mostly larvae, but sometimes eggs and rarely pupae) were collected and transferred to the insectarium room (27°C with 75% of relative humidity) for raising, emergence and adult stage identification. The number of larvae and pupae (identified to the genus level) was also recorded. Once emerged, adult mosquitoes were killed at -20°C and identified using the appropriate adult mosquito determination key (for references, see the list in Maquart *et al.*, 2021b).

### Environmental analysis

We acquired daily rainfall estimates from the Climate Hazards Group InfraRed Precipitation with Station data (CHIRPS: https://www.chc.ucsb.edu/data/chirps), calculated from rain gauge and satellite observation at a 0.05° spatial resolution (about 5.4 km at the latitude of Phnom Penh). We were thus able to calculate the rainfall for each day of capture as well as the cumulative rainfall for the 7-, 14-, 21- and 28-days preceding captures.

In order to produce a fine mapping of land use/land cover (LULC), we requested a very high spatial resolution satellite image through the French DINAMIS and GEOSUD programs, which facilitate access to such images for research. We obtained a SPOT7 (Airbus DS 2019) satellite image acquired on the 11^th^ of December 2019 (through the agreement DINAMIS Id 2020–111-Sci) with a 1.5-meter spatial resolution in panchromatic mode and 6 meters in multispectral mode. We conducted an object-based image analysis with eCognition software (eCognition Developer 9.0.3), which involves a first step of segmentation of the image into objects according to the radiometric properties of the pixels, followed by a classification step of these objects according to their properties (color, texture, size, shape) and their context. We also imported the road information available from OpenStreetMap (Openstreetmap contributors), as it is the most accurate geographical information for the road network in Phnom Penh. This data helped improve a homogeneous detection of the main and small roads. In order to validate the classification, an independent person selected 50 representative locations for each LULC class. This allowed to build a confusion matrix to measure the difference between the observations and the classification.

We used the LULC to describe landscapes around each pagoda, with QGIS software [20]. Without *a priori* knowledge of the distance at which landscapes could have an impact on the composition of mosquito communities in a pagoda, we chose to test the calculation of landscape indices at different distances (within a radius of 250, 500 or 1000 meters from the centroid of each pagoda indicated in S1 Table) and see which indices will be most important in the statistical analyses. A short distance of 250 meters will describe the immediate environment of the pagodas, while longer ones (500 or 1000 meters) may reveal the presence of other land uses such as herbaceous vegetation, crops or water. In each buffer zone, we calculated several indices: the proportion of surface covered by each class (noted LSPP for Landscape proportion), the patch density (the number of patches of each class per hectare of buffer zone, noted PD), and the edge density (the perimeter of all the patches of each class, noted ED, in meters per hectare). These last two indices (patch and edge densities) reflect the state of the landscape fragmentation. We calculated LSPP indices using QGIS software and both PD and ED indices using the “landscapemetrics” package [21] in R 4.2.1, a language and environment for statistical computing [22].

### Statistical analysis

We performed all statistical analyses using R 4.2.1 software. Statistical significance was set at p < 0.05. During the trapping weeks, some traps may have gone missing (disappeared or been knocked over). The incidents were taken into account: the actual number of traps multiplied by weeks per site was calculated to adjust the number of mosquitoes trapped per “trap-week”. Statistical analyses were performed on this adjusted number of trapped mosquitoes. We first explored the temporal correlations between the number of trapped mosquitoes of the two *Aedes* species on each date, and between each species and daily rainfall for each day of capture, or the cumulative rainfall for the 7-, 14-, 21- and 28-days preceding captures. We also explored the correlations and performed Principal Component Analyses (PCA) to assess the relationships between the number of mosquitoes of the two *Aedes* species and the environmental variables at each trapping site. In order to test multiple linear regressions, we calculated pairwise correlations between the environmental indices and removed those with the highest correlations (> 0.85 in an initial selection), as variables that are highly correlated with each other can render the models unstable [23,24]. Then a backward elimination multiple linear regression modelling was performed to see if the number of *Aedes* mosquitoes of each species (as the response variables) could be modelled based on these environmental indices (*i.e.,* the explanatory variables) at each site. The best models were selected based on the Akaike Information Criterion (AIC). For the final models, we verified the absence of multicollinearity of the variables by calculating the Variance-Inflation Factor (VIF, acceptable if the square root of VIF is less than 2) with the “performance” R package [25]. We gradually removed from the models the variables with the highest VIF (greater than 10, then greater than 5) [26]. Finally, we checked the normality of residuals and the absence of outliers with the “performance” R package.

## Results

### Trapping success throughout space and seasons

Collections were conducted in the 40 pagodas from March 2019 to February 2020. Accounting for missing traps, the number of trap-weeks per pagoda ranged from 30 (5 traps during 6 weeks) to 21 (9 traps missing in total), with an average of 25.8 (standard deviation of 2.4). The highest losses in traps were observed during the first three weeks. A total of 68,363 larvae were collected, and 9,050 adults emerged (survival rate of 13.2%): 5,080 (56.1%) were identified as *Ae. aegypti* and 2,771 (30.6%) as *Ae. albopictus*. The remaining 1,199 emerged mosquitoes belong to 8 other species: *Anopheles vagus*, *Culex brevipalpis, Cx. gelidus*, *Cx. quinquefasciatus*, *Cx. tritaeniorhynchus*, *Cx. vishnui*, *Lutzia fuscana* and *Toxorhynchites splendens*.

**Fig 2:**
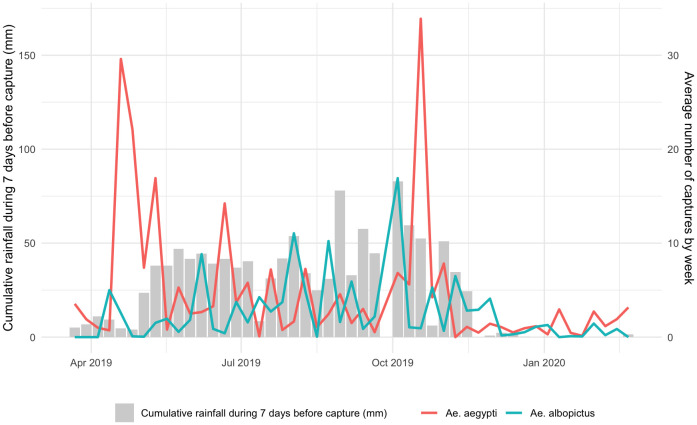
Number of *Ae. aegypti* and *Ae. albopictus* trapped by week and associated rainfall.

Temporally, the average number of emerged *Ae. aegypti* per trap per week varied between 0 and 33.9 (average of 4.8), while it varied between 0 and 16.9 (average of 2.6) for emerged *Ae. albopictus*, which were caught in lower densities (Fig 2). If the field emergence rates were consistent with what we estimated in the laboratory (85%), this would result in approximately 0 to 221.6 adults of *Ae. aegypti* per trap (average of 31.4) and 0 to 110.5 adults of *Ae. albopictus* per trap (average of 17). There was no correlation between the average number of emerged *Ae. aegypti* per trap per week and that of emerged *Ae. albopictus*. However, it should be noted that the 5 weeks during which the average number of emerged *Ae. aegypti* per trap was higher than 10 were weeks when the average number of emerged *Ae. albopictus* was very low. These weekly variations showed a period between 19 April and 10 May 2019 when *Ae. aegypti* catches were particularly high. The peak of captures was recorded on 18 October at an isolated date. No relationship was found between rainfall and the number of *Ae. aegypti.* On the other hand, the captures of *Ae. albopictus* were strongly correlated (p < 0.01) with the amount of rainfall that preceded the captures, regardless of the duration of the period (7, 14, 21 or 28 days) (Fig 2).

**Fig 3:**
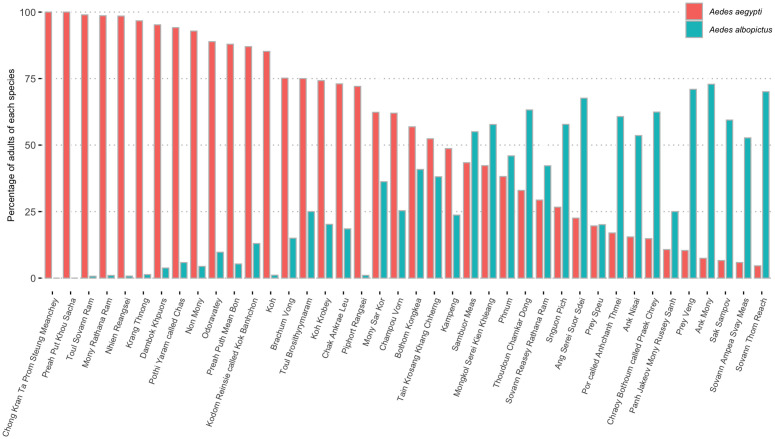
Percentage of *Ae. aegypti* and *Ae. albopictus* trapped in each pagoda (pagodas are ordered by a decreasing percentage of *Ae. aegypti*).

Spatially, *Ae. aegypti* was found in the 40 sampled pagodas and *Ae. albopictus* in 38 sites. The percentage of *Ae. aegypti* among adult mosquitoes by pagoda was negatively and significantly correlated to the percentage of *Ae. albopictus* (p < 0.001) (Figs 3 and S2).

### Environmental patterns of mosquito distribution

The final Land Use/Land Cover (LULC) map includes nine classes (1. Roads, 2. Built-up areas, 3. Water bodies and rivers, 4. Wetlands, 5. Dry bare areas, 6. Bare crop fields, 7. Low vegetation areas, 8. High vegetation areas, 9. Forested areas). The first class “1. Roads” was based on OpenStreetMap data and was removed from the accuracy calculation. Additionally, the last class, “9. Forested areas”, represented a single patch in the south of the satellite image and was also removed from the accuracy calculation. The confusion matrix (Table 1) showed an overall accuracy of 90% and a Kappa index of 0.88, which is very satisfactory for future uses of this classification [27]. The least accurate class (precision of 69.2%) was “7. Low vegetation areas”, due to confusion with several other classes having few vegetation. All other classes exceeded a high precision of 83.3%.

**Table 1:** Confusion matrix of the Land Use/ Land Cover classification. Overall accuracy = 90%, Kappa = 0.88.

Truth data									
**LULC classes**	**2**	**3**	**4**	**5**	**6**	**7**	**8**	**Total**	**Precision (%)**
2. Built-up areas	**43**	0	0	0	0	0	0	43	100
3. Water bodies and rivers	0	**48**	4	0	0	0	0	52	92.3
4. Wetlands	0	2	**40**	0	0	0	0	42	95.2
5. Dry bare areas	0	0	0	**48**	0	0	0	48	100
6. Bare crop fields	0	0	2	2	**45**	5	0	54	83.3
7. Low vegetation areas	7	0	4	0	5	**45**	4	65	69.2
8. High vegetation areas	0	0	0	0	0	0	**46**	46	100
Truth overall	50	50	50	50	50	50	50	350	
**Producer accuracy (%)**	**86**	**96**	**80**	**96**	**90**	**90**	**92**		

In the landscape analysis of mosquito distribution, we finally removed two classes that were not present in the study area: “4. Wetlands” and “9. Forested areas”. We finally obtained a total of 63 landscape indices: 3 types of index for 3 buffer sizes and 7 LULC classes (See S2 Table. for the names, description, units and descriptive statistics of the 63 indices). We verified that the 40 randomly selected pagodas represent different levels of urban and peri-urban landscapes of Phnom Penh. In their immediate environment (250-meter radius), the pagodas have proportions of surfaces covered by roads that spread out regularly from 0% to 11.4%, with a mean of 4.2% and a standard deviation of 2.5% (S1 Fig). The proportion of surfaces covered by built-up areas also spread out regularly from 2.8% to 89.7%, with a mean of 44.8% and a standard deviation of 28.0%).

**Fig 4:**
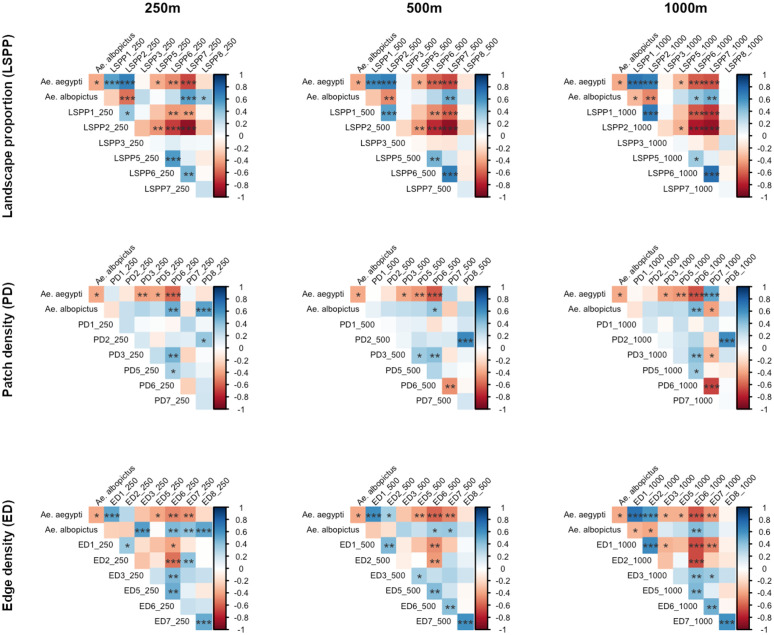
Correlation matrices between the average number of *Ae.* *Aegypti* and *Ae. albopictus* and all landscape indices. Significant correlations (* for p < 0.05, ** for p < 0.01, *** for p < 0.001) are highlighted in blue for positive *vs.* red for negative correlations, with darker colors for higher significance level.

The average number of *Ae. albopictus* showed the most significant correlations (p < 0.001) with 5 landscape indices, only at short distances (250m): positively, with both patch density and edge density related to high vegetation areas; positively with the edge density related to water bodies and rivers; positively with the proportion of low vegetation areas; and negatively with the proportion of built-up areas (correlations noted *** in Fig 4). The average number of *Ae. aegypti* showed the most significant correlations (p < 0.001) with a greater number of 21 landscape indices at different distances. The most significant correlation (p < 0.001) was found to be positive with the edge density related to roads at longer distances (1000m). This was followed by a set of 7 indices of landscape proportions related to roads (positive), built-up areas (positive) and low vegetation areas (negative) at different distances.

These very strong correlations imply that the number of mosquitoes of each species can be estimated, in a very simple way, as a linear function of a single variable. Thus, the number of *Ae. albopictus* can be assessed by the edge density related to high vegetation areas at a short distance (ED8_250, *i.e.*, dense high vegetation in a radius of 250 meters) (p < 0.001, with the formula: *Ae. albopictus* = 1.1 * ED8_250 + 41.8), while the number of *Ae. aegypti* can be assessed by the edge density of roads at long distances (ED1_1000, *i.e.,* high density of roads in a radius of 1km) (p < 0.001, with the formula: *Ae. aegypti* = 1.5 * ED1_1000 - 37.5).

**Fig 5:**
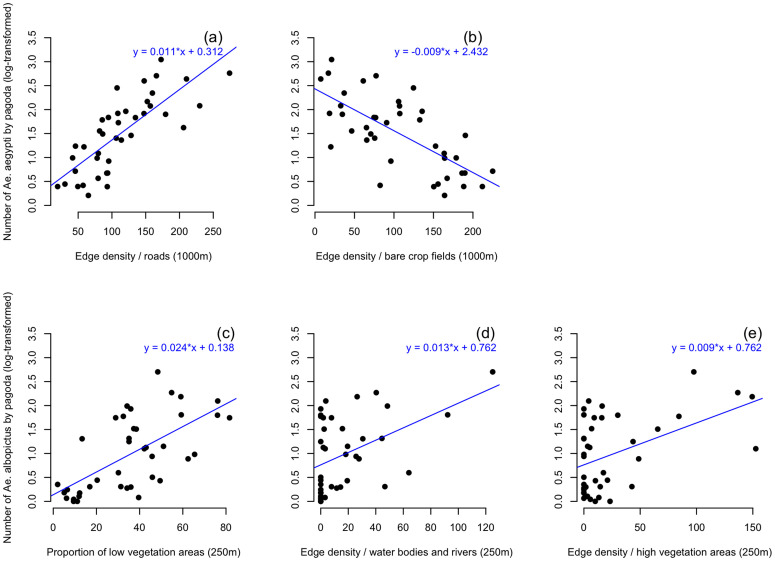
Number of mosquitoes (log-transformed) of each species by pagoda according to the best explanatory variables from the multiple linear regression, (a)* Ae. aegypti* and the edge density related to roads within 1000m buffers, (b)* Ae. aegypti* and the edge density related to bare crop fields within 1000m buffers, (c)* Ae. albopictus* and the proportion of low vegetation areas within 250m buffers, (d)* Ae. albopictus* and the edge density related to water bodies and rivers within 250m buffers, (e)* Ae. albopictus* and the edge density related to high vegetation areas within 250m buffers.

There are also strong correlations between landscape indices, as seen in the case of indices of the same nature calculated on different radii or indices linked to the same land-use class (for example, edge density and proportion of roads in 1 km buffers). Some of these highly correlated variables were removed, and the VIF was checked to avoid multicollinearity among the variables included in the models. The most parsimonious multiple linear regression model to explain the number of *Ae. aegypti* (log-transformed) only includes the edge density of roads at a distance of 1,000 meters (estimate of 0.007, p < 0.001) and the edge density of bare crop fields at a distance of 1,000 meters (estimate of -0.004, p < 0.05) (Fig 5). The most parsimonious multiple linear regression model to explain the number of *Ae. albopictus* (log-transformed) is a model with three variables (all at a short distance of 250 meters): the proportion of low vegetation areas (estimate of 0.018, p < 0.001), the edge density of water bodies and rivers (estimate of 0.007, p < 0.05), and the edge density of high vegetation areas (estimate of 0.005, p < 0.05) (Fig 5).

## Discussion

**Fig 6:**
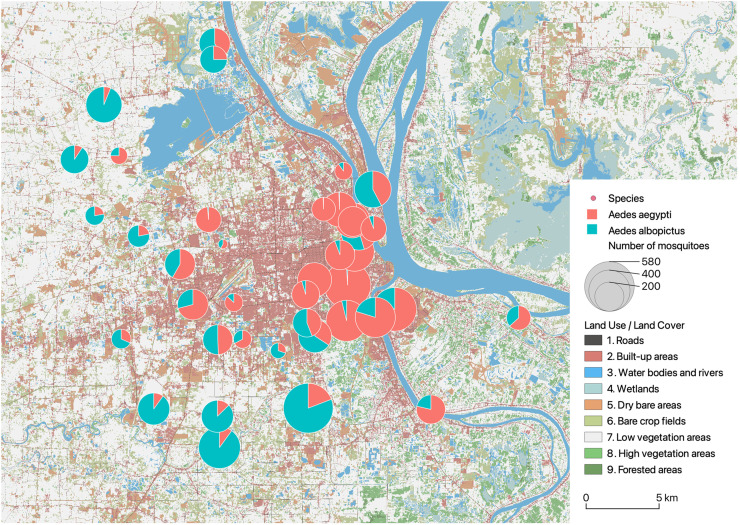
Map of the total number of *Ae. aegypti* and *Ae. albopictus* in each pagoda.

The densities of *Aedes* mosquitoes differ in space and time between the two species. *Ae. aegypti* is predominantly found in the center of Phnom Penh, while *Ae. albopictus* is more common on its periphery (Fig 6). These contrasting distributions are clearly reflected in the environmental indicators. Indeed, the strong associations between the number of *Ae. albopictus* and landscape indices at short distances highlight the importance of nearby environments with vegetation (high or low) and water, *i.e.,* in peri-urban areas. For *Ae. aegypti*, the associations with landscape indices are more numerous, underscoring the importance of highly urbanized environments. The fact that these correlations are even stronger when the observation distances are large (500–1000 meters) further shows that the presence of *Ae. aegypti* is concentrated in the heart of urban areas, far from peri-urban areas.

**Fig 7:**
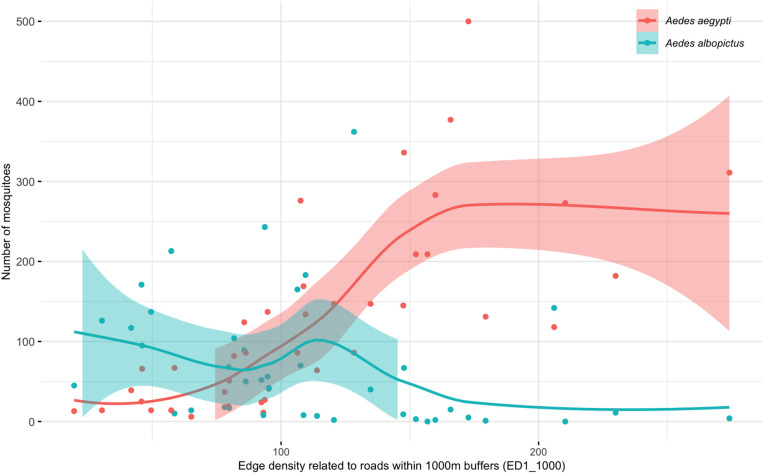
Comparison of the number of *Ae. aegypti* and *Ae. albopictus* by pagoda along a gradient of urbanization (edge density of roads in a radius of 1000 meters).

If we compare the number of mosquitoes per species along a gradient of urbanization, represented by road density (edge density of roads in a radius of 1000 meters), Fig 7 clearly shows that when road density is low (peri-urban landscapes, index <75), *Ae. albopictus* mosquitoes predominate (Fig 7). This is followed by a transition phase in which both species cohabit at low and equivalent densities. When the road network is denser (urban landscapes, index > 125), *Ae. aegypti* mosquitoes are in the majority and in large numbers, and *Ae. albopictus* mosquitoes are in smaller numbers. These distributions of the two *Aedes* species have consequences for the transmission of dengue in urban and peri-urban environments. Although the causes of dengue fever outbreaks are multi-factorial, environmental changes such as urbanization may be one of the leading factors. *Ae. albopictus* mosquitoes are strongly anthropophilic and present a higher blood-feeding rate in urban areas in Italy, where human population density is high, than that in rural areas [28], making it a more suitable vector in urban environments.

From a temporal perspective, the densities of *Ae. albopictus* are associated with rainfall in the weeks prior to the capture of larvae, showing a strong correlation with the presence of water in the environment. As a result, *Ae. albopictus* populations are likely to be more sensitive to climatic disturbances and induced abnormal events (e.g., floods, droughts) (Bonnin et al. 2022). In contrast, the lack of association between *Ae. aegypti* and rainfall reflects its disconnection from seasonal variation in highly anthropized environments. *Ae. aegypti* populations are expected to be less sensitive to climatic disturbances (Doeurk et al. 2024).

Considering that *Ae. albopictus* is a competent vector for all four dengue serotypes and can transmit at least 22 arboviruses [29], its presence in the capital could lead to serious arbovirus outbreaks. Also, given their different seasonal dynamics, both species may have complementary roles in maintaining the virus across different seasons. Consequently, it is important to consider the ecology of *Ae. albopictus* alongside that of *Ae. aegypti* when developing vector and disease control programs in Phnom Penh. This study is therefore important to the Ministry of Health in Cambodia as it provides evidence of the need to increase surveillance and control of this species in suburban and rural areas.

*Ae. albopictus*, originally from the forests of Southeast Asia where it was likely zoophilic (*i.e.*, feeding on wildlife), has progressively adapted to anthropogenic environments, which provided alternative blood sources (domestic animals and humans) and water collections for larval habitats [13,30]. Human migrations have favored its spread into new areas, where it quickly became an opportunistic container breeder, using either natural or artificial containers, and demonstrating the ability to survive in small water collections such as tires, plastic buckets, and plastic cups [31]. Today, it occurs in rural, suburban and urban areas [10,13].

Several studies have focused on weather conditions, specifically temperature and rainfall [32], as temperature drives mosquito reproduction, maturation and mortality [33], while rainfall influences the availability of breeding sites [34]. However, not much work has investigated the relationship between these species and landscape patterns. Spatial heterogeneity of *Ae. aegypti* was observed in Puerto Rico, where the association between temperature, precipitation and monthly changes in dengue transmission varies spatially and is associated to local climate differences [35]. In La Réunion, the spatial and temporal distribution of *Ae. albopictus* was linked to both climate (precipitation and temperature) and the productivity of breeding sites, especially anthropized ones (Boyer et al. 2014). Similar observations were made in Brazzaville (Wilson-Bahum 2000), Taiwan [15], Kuala Lumpur, Malaysia (Chen et al. 2006), Singapore city (Chan et al. 1971; Chung et al. 2002), Thailand (Campbell 2013; Jirakanjanakit 2007), Guangdong, China (Liu et al. 2019), and both Hanoi (Tsuzuki et al. 2013) and Ho Chi Minh (Higa et al 2010), Vietnam.

Vector surveillance is crucial for determining the distribution, population density, larval habitats, and spatiotemporal risk factors related to dengue transmission. The ovitrap surveillance system is an alternative for long-term vector surveillance, providing insight into population dynamics and the spatiotemporal distribution of mosquito vectors, which can improve dengue prevention and control programs. The ovitraps used in this study are inexpensive and easy to use, constituting an effective tool for monitoring dengue vectors [36]. They have been used for routine surveillance of dengue in several countries, such as Hong Kong [37], Singapore [38], Taiwan (Chen, 2006), and Australia [39]. Ovitrap surveying is preferable to larval surveys because it is an active surveillance method that detects not only immature mosquitoes but also eggs laid by gravid females [40,41].

This study was designed to be replicable for monitoring changes in *Aedes* mosquito populations in relation to urban landscapes changes. It could be replicated in the near future in Cambodia, where urbanization is progressing rapidly. Moreover, it provides a methodological framework that can be easily adapted to other cities, allowing for the characterization of the importance of different climatic and urban contexts in these dynamics. Despite the container-breeding habits of these species, numerous factors determine whether immatures successfully emerge as adults, including washout, desiccation, container disturbance, density-dependent mortality, food availability, and temperature effects on survival and development. While the geographical approach presented here is fully replicable, the entomological component remains open to discussion. Collecting larvae would require accurate larval identification rather than relying solely on adult proxies. We therefore suggest either targeted adult collections in pagodas or direct larval sampling followed by identification to minimize mortality and improve species-level resolution.

## Conclusions

This study confirms the presence of both *Aedes* species in the urban environment of Phnom Penh, with contrasting distributions: the dense urban core favors *Ae. aegypti*, while peri-urban areas with vegetation and water are favorable to *Ae. albopictus*. From these distributions, we cannot determine whether they are due to ecological preferences or competition between species. We also conclude that the dynamics of *Ae. albopictus* may be more affected by climatic anomalies, as its densities are linked to rainfall and the presence of nearby water sources. This study suggests that both species are highly sensitive to human-induced changes in land use and the broader impacts of global change.

## Supporting information

S1 Table List of the pagodas. (XLSX)

S2 Table Description of the landscape indices. (XLSX)

S1 Fig Proportion of surface covered by (a) roads () and by (b) built-up areas in the immediate environment of each pagoda (in a radius of 250 meters). (TIFF)

S2 Fig Percentage of *Ae. aegypti* and *Ae. albopictus* by pagoda. (TIFF)
