# Supplementary figures and images for: Spatio-temporal distribution and environmental determinants of dengue vectors in Phnom Penh, Cambodia
Source: PLoS Negl Trop Dis. 2025 Oct 29;19(10):e0013667. doi: 10.1371/journal.pntd.0013667 (PMC12582508; doi:10.1371/journal.pntd.0013667)

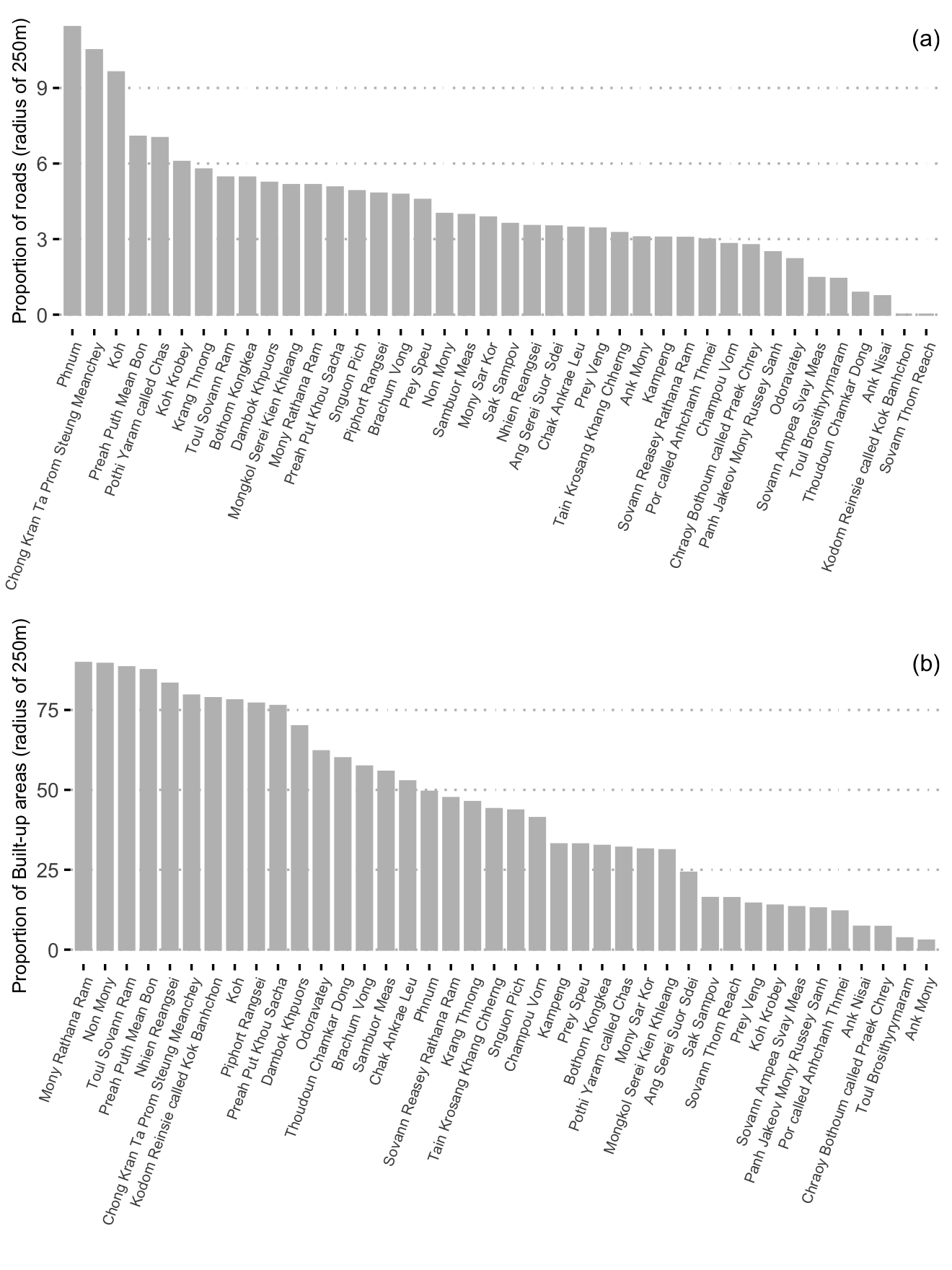

Supplement: S1 Fig — (TIFF) [file pntd.0013667.s003.tiff]

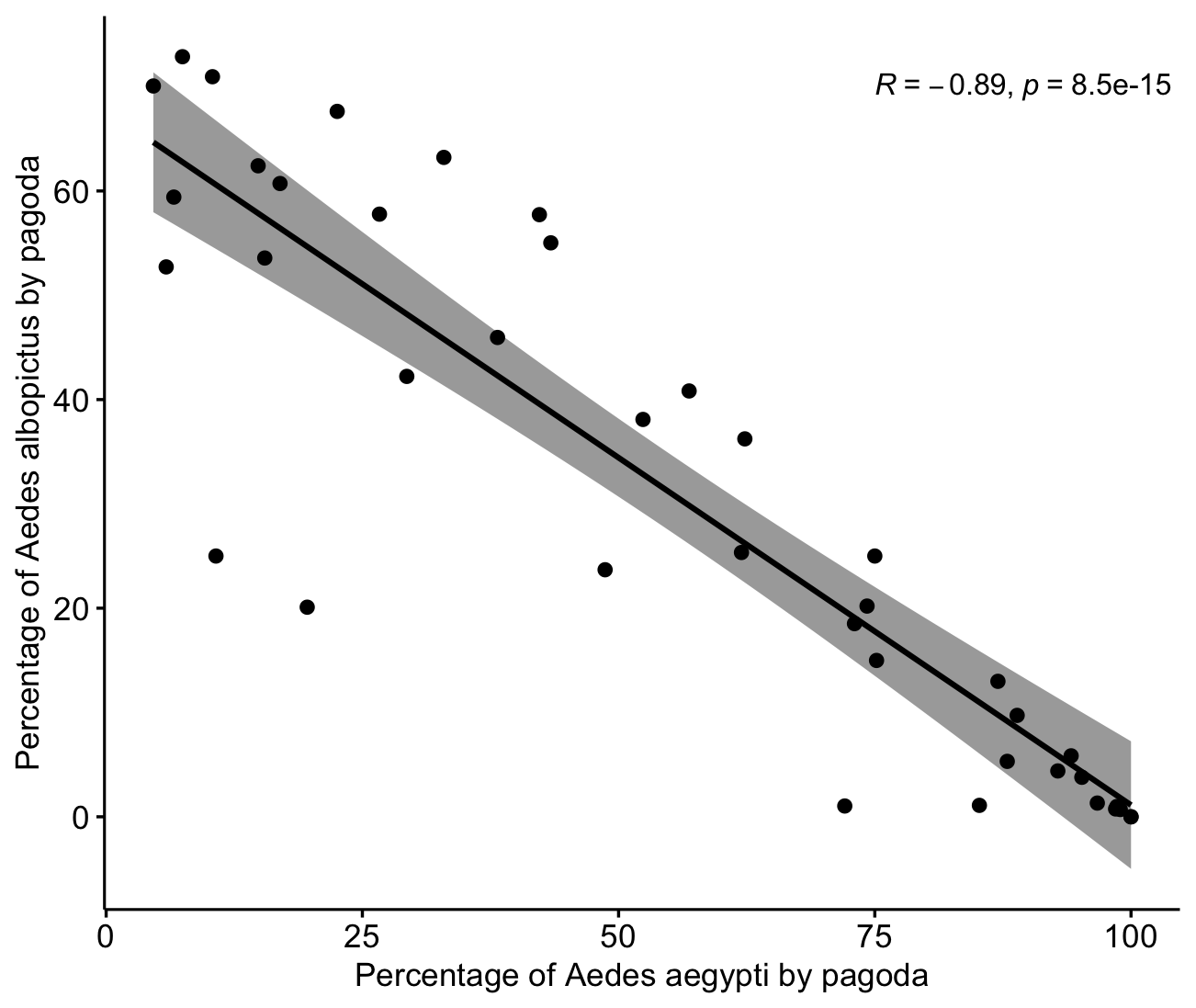

Supplement: S2 Fig — (TIFF) [file pntd.0013667.s004.tiff]
